# The Rectum and Anus: Their Diseases and Treatment

**Published:** 1887-12

**Authors:** 


*The Rectum and Anus: their Diseases and Treatment.* By CHAS. B. BALL, F.R.C.S.I. Pp. 410. London: CASSELL & CO.

We have been most favourably impressed by this work. It is quite one of the best of the excellent series of *Clinical Manuals* now being issued by Messrs. Cassell and Co. As a full, clear, and trustworthy description of the diseases which it deals with, it is certainly second to none in the language. The author is evidently well read in the literature of the subject; and has nowhere failed to describe what is best up to date. But what pleases the reader most is the keen discrimination with which the author handles novel modes of treatment; bringing to bear on their consideration a mind that is well trained, not only in logical method, but in general and special experience. One of the best chapters in the book, that on Colotomy, is an example of this. The author clearly sees and appreciates the value of the resuscitated operation of laparo-colotomy, and has devised sensible and ingenious improvements in the operation; but he is not carried away either by his own reasoning or his own innovations—as so many perfervid geniuses are—but clearly and faithfully describes the advantages of the usual operation. This is but an isolated example of the whole book, which we look upon as a model of what such a work should be.

